# Power Comparisons and Clinical Meaning of Outcome Measures in Assessing Treatment Effect in Cancer Cachexia: Secondary Analysis From a Randomized Pilot Multimodal Intervention Trial

**DOI:** 10.3389/fnut.2020.602775

**Published:** 2021-01-14

**Authors:** Trude R. Balstad, Cinzia Brunelli, Caroline H. Pettersen, Svanhild A. Schønberg, Frank Skorpen, Marie Fallon, Stein Kaasa, Asta Bye, Barry J. A. Laird, Guro B. Stene, Tora S. Solheim

**Affiliations:** ^1^Department of Clinical and Molecular Medicine, Faculty of Medicine and Health Sciences, NTNU–Norwegian University of Science and Technology, Trondheim, Norway; ^2^Cancer Clinic, St. Olavs Hospital, Trondheim University Hospital, Trondheim, Norway; ^3^Palliative Care, Pain Therapy and Rehabilitation Unit, Fondazione IRCCS Istituto Nazionale dei Tumori, Milan, Italy; ^4^Edinburgh Cancer Research Centre (IGMM), University of Edinburgh, Edinburgh, United Kingdom; ^5^Department of Oncology, European Palliative Care Research Centre (PRC), Oslo University Hospital and Institute of Clinical Medicine, University of Oslo, Oslo, Norway; ^6^Department of Nursing and Health Promotion, Faculty of Health Sciences, OsloMet–Oslo Metropolitan University, Oslo, Norway; ^7^St. Columba's Hospice, Edinburgh, United Kingdom; ^8^Institute of Genetics and Molecular Medicine, University of Edinburgh, Edinburgh, United Kingdom

**Keywords:** cachexia, multimodal management, outcome measures, biomarkers, body composition, effect size, sample size (*n*)

## Abstract

**Background:** New clinical trials in cancer cachexia are essential, and outcome measures with high responsiveness to detect meaningful changes are crucial. This secondary analysis from a multimodal intervention trial estimates sensitivity to change and between treatment effect sizes (ESs) of outcome measures associated with body composition, physical function, metabolism, and trial intervention.

**Methods:** The study was a multicenter, open-label, randomized pilot study investigating the feasibility of a 6-week multimodal intervention [exercise, non-steroidal anti-inflammatory drugs, and oral nutritional supplements containing polyunsaturated fatty acids (*n*−3 PUFAs)] vs. standard cancer care in non-operable non-small-cell lung cancer and advanced pancreatic cancer. Body composition measures from computerized tomography scans and circulating biomarkers were analyzed.

**Results:** Forty-six patients were randomized, and the analysis included 22 and 18 patients in the treatment and control groups, respectively. The between-group ESs were high for body weight (ES = 1.2, *p* < 0.001), small for body composition and physical function [handgrip strength (HGS)] measures (ES < 0.25), moderate to high for n-3 PUFAs and 25-hydroxyvitamin D (25-OH vitamin D) (ES range 0.64–1.37, *p* < 0.05 for all), and moderate for serum C-reactive protein (ES = 0.53, *p* = 0.12). Analysis within the multimodal treatment group showed high sensitivity to change for adiponectin (ES = 0.86, *p* = 0.001) and n-3 PUFAs (ES > 0.8, *p* < 0.05 for all) and moderate for 25-OH vitamin D (ES = 0.49, *p* = 0.03). In the control group, a moderate sensitivity to change for body weight (ES = −0.84, *p* = 0.002) and muscle mass (ES = −0.67, *p* = 0.016) and a high sensitivity to change for plasma levels of 25-OH vitamin D (ES = −0.88, *p* = 0.002) were found.

**Conclusion:** Demonstrating high sensitivity to change and between treatment ES and body composition measures, body weight still stands out as a clinical and relevant outcome measure in cancer cachexia. Body composition and physical function measures clearly are important to address but demand large sample sizes to detect treatment group differences.

**Trial registration:** ClinicalTrials.gov identifier: NCT01419145.

## Introduction

Cancer cachexia is a complex multifactorial syndrome resulting in progressive weight loss due to loss of skeletal muscle mass with or without depletion of adipose tissue, leading to progressive loss of physical function (1). Discussion of how to evaluate the effect of any anti-cachexia therapy is continuously ongoing, and there is no consensus as to the optimal outcome measures in clinical trials (2, 3). Weight loss is the defining factor of cachexia according to the international cachexia definition but may not always be a valid indicator (2). Weight gain might be due to edema and/or ascites and may conceal muscle loss due to adiposity. Change in lean body mass is regularly used as an outcome measure in clinical trials, but the magnitude of clinically relevant changes has not yet been established. The loss of lipid reserves may also contribute to the cachexia phenotype. Depletion of fat depots is more prominent and often precedes loss of muscle mass in cancer patients (4, 5), but the significance of fat mass as an outcome measure in cachexia trials is not well-studied. Candidate outcome measures should be responsive to change, which implies that they need to be specific to the cachexia pathophysiology. Ideally, such outcome measures should not be significantly influenced by other factors contributing to wasting, such as antineoplastic therapy or immobilization. Nevertheless, this is practically impossible as the cachexia pathophysiology is complex, and any cachexia treatment may be influenced by effects of antineoplastic treatment, as treating cancer is also a treatment for cachexia.

The clinical need for early diagnosis and treatment of cachexia supports the need to identify specific biomarkers that precociously detect the wasting process (6). If cachexia intervention trials can demonstrate beneficial effects on body composition measures, an important question is whether circulating biomarkers representing key metabolic alterations can be used complementary to such clinical outcomes and add information about the underlying pathophysiology. So far, a limited number of clinical outcome measures have been explored in cachexia trials, most likely a consequence of ongoing definitional ambiguities together with the complexity of the condition. There is a need to establish reliable clinical outcomes, including circulating biomarkers, and evaluate their sensitivity to change in patients with cancer cachexia.

This report presents secondary analyses of data from a randomized phase II multimodal intervention trial for the treatment of cachexia evaluating implementation and effect of oral nutritional supplements (ONSs) containing polyunsaturated fatty acids (*n*−3 PUFAs), exercise, and non-steroidal anti-inflammatory drugs (NSAIDs) compared to standard cancer care (7). The multimodal intervention resulted in a stabilization of body weight, while patients in the control arm lost weight (7). The overall aim of the present study was to estimate sensitivity to change and between treatment effect sizes (ESs) of outcome measures associated with body composition, physical function, metabolism, as well as markers of the trial intervention. Considering these outcome measures, implications for trial design with regard to sample size will be discussed.

## Materials and Methods

### Trial Design and Patients

The study was a multicenter, open-label, pilot randomized phase II study investigating the feasibility of a 6-week multimodal intervention for cachexia vs. standard cancer care. This study recruited those with non-operable non-small-cell lung cancer (NSCLC) (stage III–IV) or advanced pancreatic cancer starting antineoplastic therapy (7). The primary aim of the feasibility study was to assess recruitment, compliance, and contamination in the control arm (7), and a phase III efficacy study is now ongoing (MENAC Trial, ClinicalTrials.gov: NCT02330926) (8). Forty-six patients were included in the study; three patients in each group were excluded due to missing blood samples at week 6. The present analysis includes 22 and 18 patients in the treatment and control groups, respectively (7). Characteristics of the study participants indicate that the two groups were comparable at baseline in terms of gender, age, cancer type, Karnofsky performance score, body mass index (BMI), and pre-inclusion weight loss (Table 1). The protocol received ethics and medical agency approval from all centers, and written informed consent was obtained from all patients. The study is registered at ClinicalTrials.gov (NCT01419145).

**Table 1:** Baseline characteristics.

	**Treatment group**	**Control group**
	**(*n* = 22)**	**(*n* = 18)**
Gender, male (*n*)	14	10
Age (years)	60 (8)	60 (9)
**Cancer type (*****n*****)**		
Pancreas stage III	4	4
Pancreas stage IV	5	5
NSCLC stage III	2	2
NSCLC stage IV	12	7
Karnofsky performance status (score)	87 (11)	87 (8)
Body mass index (kg/m^2^)	24 (4.4)	23.9 (2.4)
**Weight loss last 6 months (*****n*****)**		
≥10%	7	4
≥5–10%	5	6
0–5%	5	4
Weight gain	1	2
Stable weight	4	2

*Data are given as mean (SD). n, indicates number of individuals; NSCLC, non-small-cell lung cancer*.

### Body Composition Measures

Anthropometric measurements for body weight (kg) and height (cm) were obtained from all participating patients, and BMI was calculated (kg/m^2^). Total muscle mass and adipose tissue area were quantified using computerized tomography (CT) imaging covering the abdomen area at the third lumbar vertebra (L3) taken at baseline and after 6 weeks (9, 10). Axial images were selected out and analyzed using the Automatic Body composition Analyzer using Computed tomography image Segmentation (ABACS) software (11). Adipose tissue cross-sectional areas were calculated using standard Hounsfield unit (HU) thresholds of −150 to −50 HU for visceral adipose tissue, −190 to −30 HU for subcutaneous adipose tissue, and −29 to +150 HU for muscle tissue (12, 13). Tissue cross-sectional areas (cm^2^) were calculated by adding up the given tissue pixels and multiplying by the pixel surface area. Visceral and subcutaneous adipose tissue cross-sectional areas were summarized to estimate total adipose tissue areas. The total muscle and adipose area were normalized for patient height to calculate total muscle and adipose index (cm^2^/m^2^).

### Physical Function

Handgrip strength (HGS) (kg) was collected at baseline and after 6 weeks and measured with a hydraulic handheld dynamometer (JAMAR). The test was performed using the dominant hand, and three test trials were performed (7, 14).

### Collection, Storing, and Processing of Biological Samples

Baseline samples were collected before the start of chemotherapy and at endpoint (week 6 ± 1 week allowed according to the protocol). C-reactive protein (CRP) was collected using standard analytical methods applied by local hospitals. Blood samples from ethylenediaminetetraacetic acid (EDTA) containers for isolation of plasma and container without additive for isolation of serum were centrifuged at 2,200 g for 10 min, aliquoted to cryotubes, and stored at −80°C. During blood sample analysis, researchers were blinded to both the sample randomization results and clinical data. All samples were analyzed in duplicate, and a fresh aliquot was used for each analysis with no prior freeze–thaw cycles.

### Analysis of Adiponectin, Zink-α2 Glycoprotein, Insulin-Like Growth Factor 1, Glycerol, and Lipolysis

Plasma levels of adiponectin, zink-α2 glycoprotein (ZAG), and insulin-like growth factor 1 (IGF-1) were measured using ELISA (R&D Systems, Abingdon, UK). A standard concentration curve was made for each ELISA plate with the manufacturer's control solution and used to calculate plasma concentrations in the samples assayed. A coefficient of variability among sample replicates calculated by dividing the standard deviation (SD) by the mean of the set of measurements expressed as a percentage of variation to the mean below 0.10 was determined to be acceptable. Glycerol was measured calorimetrically from serum in μmol/L concentrations (Lipolysis kit LIP-3-NC, Zen-Bio, Durham, NC, USA). Lipolysis is presented as glycerol umol/L/total adipose index (cm^2^/m^2^) (15).

### Plasma *n*−3 Polyunsaturated Fatty Acids and 25-Hydroxyvitamin D Analysis

Phospholipids (PLs) from blood plasma were extracted and fatty acids from the PL were transmethylated with boron-tri-fluoride in methanol. Quantification of *n*−3 PUFAs [eicosapentaenoic acid (EPA), docosahexaenoic acid (DHA), docosapentaenoic acid (DPA)] from PL was performed using gas chromatography. The quantification is based on the use of an internal standard with known concentration and the instrument Agilent 6890N gas chromatograph with GC ChemStation software was used. PL concentration of *n*−3 PUFAs was calculated as % of total fatty acids in plasma PL. Plasma levels of 25-hydroxyvitamin D (25-OH vitamin D) were measured based on an ultra-performance liquid chromatography technique and detection by tandem mass spectrometry [Acquity UPLC® I Class med Xevo TQS MSMS (Waters)]. This assay measures both 25-OH calcidiol (vitamin D_3_) and 25-OH calciferol (vitamin D_2_), and the sum of these two are presented. Both *n*−3 PUFAs and 25-OH vitamin D analyses were done at the Department of Medical Biochemistry, St. Olavs University Hospital, Trondheim, Norway.

### Statistics

Descriptive statistics are presented as means and SDs. All analyses were carried out on the modified intention-to-treat population (defined as all randomized patients with both baseline and week 6 assessments). Comparisons between groups were conducted using *t*-tests for independent samples, while paired sample *t*-tests were used to evaluate changes within each study group. For each outcome, ESs within and between groups (ES_WG_ and ES_BG_) were calculated using appropriate formulas. ES_WG_ was calculated using Cohen's d for one-sample pre–post design to estimate sensitivity to change over time in each treatment group separately (16). Positive and negative values of ES_WG_ indicate, respectively, an increase and a decrease in the outcome over time. ES_BG_ was calculated using Hedges' g for two-independent sample design on the pre–post variations to estimate between treatment effects (16). A positive ES_BG_ value indicated an advantage for the treatment arm with respect to the control. Reference values for small (<0.2), medium (<0.5), and large (>0.8) ESs were used for result interpretation (17). Sample size per treatment arm by ES_BG_ of the various outcome measures was calculated by *t*-test for independent samples (alpha error = 0.05, power 0.9) and plotted in order to compare the relative power of the different outcome measures. Analyses were performed using IBM SPSS Statistic Software 25 for Windows and Stata 15.1 for Windows (StataCorp, College Station, Texas, USA).

## Results

### Body Mass and Body Composition

At baseline, the degree of weight loss was equally distributed between the two arms (Table 1). Mean (SD) change in body weight from baseline to week 6 within the two groups showed a small increase within the treatment arm [1.0 (2.5), *p* = 0.08, ES_WG_ = 0.40] and a moderate, significant decrease within the control group [−2.1 (2.5), *p* = 0.002, ES_WG_ = −0.84] (Table 2). A significant difference between the two arms was found (*p* < 0.001) with a high ES_BG_ = 1.2 (Table 2).

**Table 2:** Changes in outcome measures according to treatment group.

		**Treatment group (*n* = 22)**	**Δ**	**Post–Pre effect size WG^**1**^**	**Control group (*n* = 18)**	**Δ**	**Post–Pre effect size WG^**1**^**	**Between groups effect size BG^**2**^**	p**
**Body mass**									
Bodyweight, kg	T0	70.5 (13.6)	1.0 (2.5)	0.40	67.1 (9.8)	−2.1 (2.5)	**−0.84**	**1.2**	***p*** **<** **0.001**
	T2	71.5 (14.0)			64.9 (9.9)				
		p* = 0.08			p* = **0.002**				
**Body composition**^a^									
Visceral adipose area (VAT) cm^2^	T0	108.4 (67.6)	0.4 (26.2)	0.02	99.9 (65.2)	−5.1 (19.4)	−0.26	0.22	*p* = 0.53
	T2	108.8 (66.1)			94.9 (55.9)				
		p* = 0.95			p* = 0.37				
Subcutaneous adipose area (SAT) cm^2^	T0	182.3 (114.5)	−5.9 (36.7)	−0.16	160.6 (70.7)	−11.2 (28.7)	−0.39	0.15	*p* = 0.67
	T2	176.4 (108.5)			149.4 (64.5)				
		p* = 0.51			p* = 0.19				
Ratio VAT/SAT	T0	0.7 (0.6)	0.03 (0.3)	0.10	0.7 (0.5)	−0.03 (0.2)	−0.15	0.25	*p* = 0.48
	T2	0.7 (0.5)			0.7 (0.4)				
		p* = 0.66			p* = 0.48				
Total adipose area, cm^2^	T0	290.7 (154.0)	−5.5 (56.7)	−0.10	260.5 (99.9)	−16.3 (39.1)	−0.42	0.21	*p* = 0.56
	T2	285.2 (149.5)			244.3 (93.7)				
		p* = 0.69			p* = 0.16				
Total adipose index, cm^2^/m^2^	T0	99.5 (52.7)	−2.1 (19.8)	−0.11	93.3 (36.5)	−5.9 (14.0)	−0.42	0.21	*p* = 0.56
	T2	97.4 (51.2)			87.4 (34.2)				
		p* = 0.65			p* = 0.16				
Skeletal muscle mass index, cm^2^/m^2^	T0	45.9 (8.9)	−1.0 (3.8)	−0.26	45.7 (8.6)	−1.8 (2.7)	−**0.67**	0.26	*p* = 0.42
	T2	45.0 (9.2)			43.9 (9.4)				
		p* = 0.19			p* = **0.016**				
**Physical function**^b^									
Handgrip strength (kg)	T0	35.6 (11.2)	−0.6 (7.1)	−0.08	32.3 (12.5)	−0.8 (5.0)	−0.17	0.03	*p* = 0.93
	T2	35.1 (9.8)			31.5 (12.4)				
		p* = 0.72			*p* = 0.55				
**Biological mediators**									
CRP (mg/dl)^c^	T0	31.8 (32.3)	−14.1 (37.9)	0.37	15.5 (21.5)	2.6 (19.6)	−0.13	**0.53**	*p* = 0.12
	T2	17.7 (26.0)			18.1 (25.8)				
		p* = 0.14			p* = 0.62				
Adiponectin (μg/ml)	T0	11.5 (4.3)	1.2 (1.4)	**0.86**	10.0 (3.9)	1.6 (2.9)	**0.55**	0.16	*p* = 0.63
	T2	12.7 (4.6)			11.6 (3.5)				
		p* = **0.001**			p* = **0.04**				
ZAG (μg/ml)	T0	55.1 (30.4)	1.1 (16.5)	0.07	47.5 (23.7)	1.4 (12.1)	0.12	0.02	*p* = 0.96
	T2	56.3 (26.5)			48.8 (24.1)				
		p* = 0.75			p* = 0.64				
IGF-1 (nmol/L)^d^	T0	20.1 (8.0)	0.5 (5.8)	0.09	16.6 (7.9)	0.3 (7.0)	0.04	0.03	*p* = 0.94
	T2	20.5 (8.0)			16.9 (8.7)				
		p* = 0.70			p* = 0.84				
Glycerol μmol/L^e^	T0	149.9 (67.7)	−0.2 (63.7)	−0.003	148.7 (78.4)	12.4 (97.6)	0.13	0.15	*p* = 0.63
	T2	149.7 (63.1)			161.1 (79.6)				
		p* = 0.99			p* = 0.61				
Lipolytic activity^f, g^	T0	1.8 (2.0)	0.8 (3.0)	0.27	1.7 (2.1)	0.8 (2.6)	0.31	−0.0006	*p* = 0.99
	T2	2.6 (4.7)			2.5 (4.6)				
		p* = 0.27			p* = 0.30				
**Nutrient components**									
EPA (% of total fatty acids in plasma PL)	T0	1.6 (0.9)	2.1 (2.2)	**0.95**	1.1 (0.4)	0.6 (0.8)	**0.75**	**0.86**	***p*** **=** **0.006**
	T2	3.7 (2.3)			1.7 (0.8)				
		p* < **0.001**			p* = **0.009**				
DHA (% of total fatty acids in plasma PL)	T0	4.3 (1.3)	1.1 (1.3)	**0.85**	4.6 (1.7)	0.4 (1.0)	0.40	**0.64**	***p*** **=** **0.046**
	T2	5.5 (1.8)			5.0 (2.0)				
		p* = **0.001**			p* = 0.13				
DPA (% of total fatty acids in plasma PL)	T0	1.0 (0.3)	0.6 (0.7)	**0.86**	0.9 (0.2)	0.1 (0.2)	**0.50**	**0.97**	***p*** **=** **0.002**
	T2	1.6 (0.8)			1.0 (0.2)				
		p* = **0.001**			p* = 0.069				
25(OH)D (nmol/L)	T0	36.1 (20.0)	3.6 (7.4)	0.49	44.9 (25.4)	−7.5 (8.5)	**−0.88**	**1.37**	***p*** **<** **0.001**
	T2	39.7 (20.5)			37.4 (20.3)				
		p* = **0.03**			p* = **0.002**				

Data are given as mean (SD); n, indicates number of individuals; T0, baseline; T2, week 6; Δ, differences between T0 and T2; CRP, C-reactive protein; PL, phospholipid; EPA, eicosapentanoic acid; DHA, docosahexanoic acid; DPA, docosapentaenoic acid; ZAG, zink-α2 glycoprotein; IGF-1, insulin-like growth factor 1; 25(OH)D, 25-hydroxyvitamin D; p* within groups between T0 and T2. Paired-sample T-Test; p** Δ between groups. Student T test;

a *n = 18 in treatment group, n = 13 in control group for adipose tissue variables, n = 17 in control and n = 22 in treatment arm for muscle mass index*;

b *n = 22 in treatment group, n = 17 in control group*;

c *n = 15 in control group, n = 17 in the treatment group*;

d *n = 21 in treatment group*;

e *n = 17 in control group*;

f *indirect in vivo lipolytic activity was assessed by serum glycerol (μmol/L) divided by total adipose index (cm^2^/m^2^)*;

g *n = 12 in control group, n = 18 in the treatment group*;

1 *Cohen's d for one-sample pre–post design*;

2 *Hedges's g for two-independent sample design; WG, within groups; BG, between groups. Bold values indicate statistic significant changes or moderate to high effect sizes*.

**Figure 1:**
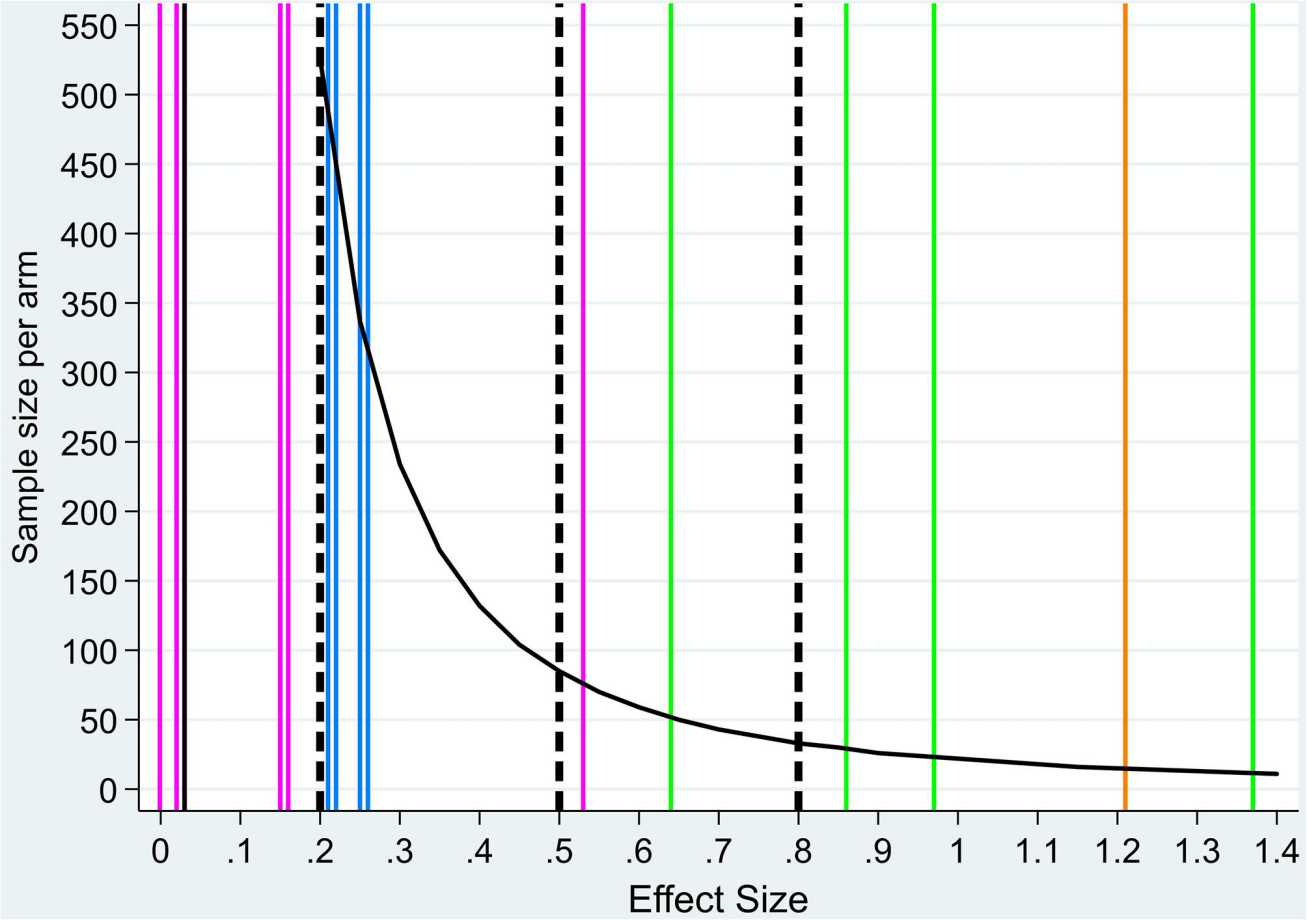
Sample size per treatment arm by effect size values. Sample size by treatment arm by effect size (ES) values (black curve). Dashed vertical lines indicate reference value for small (<0.2), medium (<0.5), and large (>0.8) ESs (17). Colored vertical lines indicate ES_BG_ for each outcome measure: body weight (orange, *n* = 1), body composition (blue, *n* = 6, two overlap, one overlaps with metabolism outcome), physical function (black, *n* = 1), metabolic mediators (pink, *n* = 6, two overlap), and nutrient components (green, *n* = 4) (exact values are reported in Table 2). Sample size values for ES < 0.2 are higher than 1,000 and not shown in the figure.

When analyzing body composition measures (Table 2), significant time change was found for skeletal muscle mass index, which decreased within the control group (−1.8 cm^2^/m^2^, *p* = 0.016, ES_WG_ = −0.67; Table 2). Most ES_WG_ in both groups were negative, indicating a decline from baseline to week 6, but these were very small in absolute magnitude within the treatment group (range −0.26 to +0.10) and higher in the control group (range −0.67 to −0.15). All ES_BG_ indicate small effects in favor of the treatment group (all below 0.26 and none of them statistically significant; penultimate column, Table 2). The sample size needed to detect ES_BG_ as those observed for body weight would be 15 participants with completed outcome measures per arm (orange color line in Figure 1), and in comparison, ~300–900 participants per arm for body composition measures (blue lines in Figure 1; sample sizes not shown for ES_BG_ < 0.2).

### Physical Function

Physical function measured using HGPs showed no significant change between the two groups (*p* = 0.93) with a very low ES_BW_ = 0.03. Within group analysis, a small mean (SD) reduction in HPS of −0.6 (7.1) (ES_WG_ = −0.08) for the treatment group and −0.8 (5.0) (ES_WG_ = −0.17) for the control group was found. Sample size by ES for HGS would be >1,000 per treatment arm (black horizontal line in Figure 1; sample sizes not shown for ES < 0.2).

### Biological Mediators

As for serum CRP levels, a nonsignificant decrease was found within the treatment group with a mean (SD) of −14.1 (37.9), medium ES_WG_ = 0.37, *p* = 0.14 (Table 2). Within the control group, a low nonsignificant mean (SD) increase of 2.6 (19.6), ES_WG_ = −0.13, *p* = 0.53, was observed with a medium ES_BG_ (0.53) in favor of the treatment group when comparing the two groups (*p* = 0.12). For CRP, sample size by ES would be 75 participants per treatment arm (blue color line in Figure 1). Plasma levels of adiponectin increased significantly within both groups from baseline to week 6 with a mean (SD) change of 1.2 (1.4) μg/ml, *p* = 0.001, with a high ES_WG_ = 0.86 for the treatment group and 1.6 (2.9) μg/ml, *p* = 0.04, and moderate ES_WG_ = 0.55 for the control group (Table 2). No significant differences in change of adiponectin levels between the groups were observed (*p* = 0.63), low ES_BG_ = 0.16. No significant change within groups or between groups were found for plasma levels of ZAG, IGF-1, glycerol, or lipolysis (Table 2) (*p* > 0.05 for all). ES_WG_ for ZAG, IGF-1, glycerol, and lipolysis in both arms were very small (<0.20), indicating no change from baseline to week 6. For adiponectin, a large ES_WG_ in the treatment arm (>0.80) and a medium ES_WG_ in the control arm (>0.50) was observed. The ES_BG_ for all variables were very small [all <0.20 in favor of the treatment arm except for lipolysis (−0.001)]. Sample sizes by ESs as those observed for adiponectin, ZAG, glycerol, or lipolysis would consequently range from around 1,000 or more participants per treatment arm (pink lines in Figure 1; sample sizes not shown for ES < 0.2).

### Nutrient Components

The recommended intake of *n*−3 PUFA containing ONS in the treatment group was two containers/day; however, the actual mean (SD) intake among the 22 patients was 1.1 (0.73) containers (range 0–2 containers/day) (7). Changes in plasma level (% of total fatty acids in plasma PL) from baseline to week 6 for EPA, DHA, and DPA are shown in Table 2. In the treatment group, significant mean (SD) increase for EPA [2.1% (2.2%), *p* < 0.001], DHA [1.1% (1.3%), *p* = 0.001], and DPA [0.6% (0.7%), *p* = 0.001] was demonstrated. In the control group, a significant increase was observed for EPA [0.6% (0.8%), *p* = 0.009]. Mean (SD) changes in EPA, DHA, and DPA from baseline to week 6 were statistically significantly increased in the treatment group compared to the control group (Table 2; *p* < 0.05 for all).

A significant mean (SD) increase of 25-OH vitamin D was observed in the treatment group [3.6 (7.4) nmol/L, *p* = 0.03] compared to a significant mean (SD) decrease in the control group [−7.5 (8.5) nmol/L, *p* = 0.03]. The change in 25-OH vitamin D levels was significant between the two groups (Table 2; *p* < 0.001). ES_WG_ for EPA, DHA, and DPA was large (>0.80 for all) and medium (0.49) for 25-OH vitamin D in the treatment arm and medium for EPA (0.75), DHA (0.40), and DPA (0.5) and large for 25-OH vitamin D (−0.88) in the control arm. The ES_BG_ were medium for DHA (0.64) and large (>0.8) for EPA, DPA, and 25-OH vitamin D in favor of the treatment arm. Accordingly, green lines in Figure 1 show that small sample sizes are needed per treatment arm for this set of variables if chosen as outcome measures (52 participants for DHA, 29 for EPA, 23 for DPA, and 12 for 25-OH vitamin D).

## Discussion

The selection of valid and useful outcome measures is a critical step when designing cancer cachexia trials. In the present study, we investigated cachexia outcome measures for their sensitivity to change and ESs between treatment groups. Outcomes investigated were related to body mass and body composition, physical function, as well as circulating biomarkers representing metabolism and the nutritional intervention. The outcome measures examined changed predominantly in favor of the treatment arm, although high ES_BG_ were demonstrated for body weight and the nutrient component biomarkers only. Furthermore, our sample size estimations show a large difference between sample sizes for body weight (*n* = 15), body composition measures (~300–900 participants) and HGS (*n* > 1,000) if used as primary outcome.

Although frequently used, body composition is a challenging primary outcome measure in cancer cachexia trials. Body composition, either measured as total lean mass (entire body weight minus fat), skeletal muscle mass, or fat mass, is in general extremely variable across the general population and in patients with cancer (18). This introduces the necessity of large sample sizes in clinical trials, which again can emphasize statistical differences that are not necessarily clinically relevant (19).

Furthermore, as a prognostic indicator, CT is considered the “gold standard” measurement providing high precision (<2% error) (20) and, demonstrating high correlation with assessment by dual-energy X-ray absorptiometry (DXA) (21). However, as an outcome measure, there are uncertainties to whether the same cross-sectional area, such as L3 level used in the present trial, captures treatment effects, especially if strength exercise intervention mainly involves large muscle groups in the upper and lower extremities (7, 8). Considering fat mass, previous studies have also reported that a single CT image slice does not accurately predict adipose tissue changes during weight loss (22). Nevertheless, compared to lean body mass measurements from DXA, muscle mass quantification from CT images yields information on a tissue-organ level reflecting striated muscle only- and skeletal muscle mass-specific changes.

Comparable trials testing the effect of novel anti-cachexia drugs [e.g., anamorelin or selective androgen receptor modulators (SARMs)] have used body composition measurement such as lean body mass (total or appendicular) as outcome measure (23–25). Different methodologies make comparison of ES_BG_ for body composition across trials challenging, and furthermore, there is an abundance of well-validated outcome measures for this purpose. Recent trials have added measures that capture changes in physical function in conjunction with skeletal muscle mass to test the efficacy of anti-cachexia treatments. Albeit endorsed by regulatory authorities, the use of such co-primary endpoints has so far had limited success, as corresponding effects are not demonstrated (26). The magnitude of muscle mass loss in the control arm in this study does not evoke a corresponding reduction in HGS. Low muscle mass is associated with reduced physical function; however, the relationship is nonlinear and, likely, there is a variable impact on physical function outcomes depending on the magnitude of changes in muscle mass (14). The potential of physical function outcomes such as HGS (and other performance testing) to detect change relative to muscle/weight changes in cancer cachexia remains unclear.

Cachexia is considered a multiorgan syndrome (27), and emerging evidence suggests there is a crosstalk between adipose tissue and skeletal muscle (28). For instance, muscle wasting seems to be preceded by signals generated from inflamed and dysregulated adipose tissue, which may be present prior to detectable loss of fat mass. The use of circulating biomarkers as outcome measures in clinical trials could potentially overcome several of these challenges by representing specific metabolic pathways. In the present study, there were neither within- nor between-group changes in any fat mass compartments or for biomarkers representing loss of fat mass such as plasma levels of ZAG, glycerol, and lipolysis. This may indicate that adipose tissue biomarkers and fat mass correspond over time. It remains to be investigated whether any of these circulating biomarkers, or others not investigated in this study, demonstrates corresponding changes with body composition. Further, the prognostic and predictive value for loss of muscle mass independent of loss of adipose tissue needs further investigation.

To understand the anti-cachexic mechanisms of any intervention, it is of importance to explore how interventions act on regulators of metabolism and inflammation. The loss of muscle mass within the control group was not followed by a corresponding change in IGF-1, a strong modulator of muscle mass synthesis. The effect of the multimodal intervention might prevent loss of muscle mass by targeting systemic inflammation and thus acting anti-catabolic rather than being anabolic. This seems supported by the change in CRP in favor of the multimodal treatment with a medium ES_BG_ of 0.53.

Adiponectin is involved in the regulation of glucose and lipid metabolism and has insulin-sensitizing and anti-inflammatory properties (29). To our knowledge, this is the first study to evaluate how adiponectin corresponds to change in body weight and body composition over time as well as response to anti-cachexic treatment. The increased levels of adiponectin within the control arm might be due to weight and muscle loss, which is also shown in cross-sectional studies comparing cachexic cancer patients to non-cachexic and healthy controls (30–32). In the intervention group, the increased adiponectin levels might be a response to the intake of *n*−3 PUFAs (33, 34). Further studies investigating the role of adipokines in cancer cachexia are necessary, as the direction and clinical meaning of change are not fully outlined.

Biomarkers may in some cases be related to parts of the intervention targeting cachexia, e.g., they may provide information about contamination and compliance and might represent a relevant outcome. The nutritional intervention biomarkers (*n*−3 PUFAs and 25-OH vitamin D) yielded the largest within- and between-group ESs corresponding to intake of the ONS. The moderate increase in EPA also within the control group may be explained by contamination if patients start taking supplements or mimic parts of the intervention (7). In unblinded randomized controlled trial (RCT) designs with nutrition and exercise interventions, outcome measures of compliance, and contamination are important to be able to assess risk of bias.

In this study, we estimated sensitivity to change and between treatment ESs from a pilot study. Albeit underpowered and not designed to compare the efficacy of an intervention, pilot studies are considered legitimate to estimate sample sizes. Still, caution is advised as estimates might be biased or unrealistic due to chance factors related to the small sample size (35). Our results revealed that >300 participants were needed per arm to detect an ES of 0.2 for skeletal muscle mass index, which are numbers comparable to the numbers of participants included in other cachexia trials with lean body mass and HGS as co-primary outcomes (24). The ongoing phase III MENAC trial is powered on body weight with a moderate ES_BG_ (0.5) as main outcome including 90 completed patients per arm (8). In parallel arm RCTs, the between-group analysis is the correct analysis approach (36). In this secondary analysis, we also analyzed within-group ESs to estimate sensitivity to change of the various outcomes explored as it can be informative when choosing the most appropriate outcomes. Evaluation of the control group receiving standard care, which to a certain extent also is anti-cachexia treatment, is consequently of importance.

In conclusion, body weight remains a clinical and relevant outcome measure in cancer cachexia, as body composition measures, HGS, and some circulation biomarkers demand large sample sizes to detect differences. So far, research has not been able to demonstrate superiority for any measure of body composition or specific biomarkers, although clearly, these are important to address in order to understand the underlying pathophysiology of weight loss in cancer cachexia. Research in cancer cachexia still needs to address both testing of treatments and evaluation of relevant outcomes until an evidence-based consensus on what to measure is reached.

## Data Availability Statement

The raw data supporting the conclusions of this article will be made available by the authors, without undue reservation.

## Ethics Statement

The studies involving human participants were reviewed and approved by the Regional Committee for Medical and Health Research Ethics (Reference 2010/2620) and Norwegian Medicines Agency (Reference 11/01673-8). The patients/participants provided their written informed consent to participate in this study.

## Author Contributions

All authors listed have made substantial, direct and intellectual contribution to the work and approved it for publication.

## Conflict of Interest

The authors declare that the research was conducted in the absence of any commercial or financial relationships that could be construed as a potential conflict of interest.

## References

[1] Fearon K Strasser F Anker SD Bosaeus I Bruera E Fainsinger RL Definition and classification of cancer cachexia: an international consensus. Lancet Oncol. (2011) 12:489–95. 10.1016/S1470-2045(10)70218-7 21296615

[2] Fearon K Argiles JM Baracos VE Bernabei R Coats A Crawford J. Request for regulatory guidance for cancer cachexia intervention trials. J Cachexia Sarcopenia Muscle. (2015) 6:272–4. 10.1002/jcsm.12083 26675232 PMC4670733

[3] Laird BJA Balstad TR Solheim TS. Endpoints in clinical trials in cancer cachexia: where to start? Curr Opin Support Palliat Care. (2018) 12:445–52. 10.1097/SPC.0000000000000387 30299325

[4] Bing C. Lipid mobilization in cachexia: mechanisms and mediators. Curr Opin Support Palliat Care. (2011) 5:356–60. 10.1097/SPC.0b013e32834bde0e 21934502

[5] Tsoli M Swarbrick MM Robertson GR. Lipolytic and thermogenic depletion of adipose tissue in cancer cachexia. Semin Cell Dev Biol. (2016) 54:68–81. 10.1016/j.semcdb.2015.10.039 26529279

[6] Loumaye A Thissen JP. Biomarkers of cancer cachexia. Clin Biochem. (2017) 50:1281–8. 10.1016/j.clinbiochem.2017.07.011 28739222

[7] Solheim TS Laird BJA Balstad TR Stene GB Bye A Johns N. A randomized phase II feasibility trial of a multimodal intervention for the management of cachexia in lung and pancreatic cancer. J Cachexia Sarcopenia Muscle. (2017) 8:778–88. 10.1002/jcsm.12201 28614627 PMC5659068

[8] Solheim TS Laird BJA Balstad TR Bye A Stene G Baracos V. Cancer cachexia: rationale for the MENAC (multimodal-exercise, nutrition and anti-inflammatory medication for cachexia) trial. BMJ Support Palliat Care. (2018) 8:258–65. 10.1136/bmjspcare-2017-001440 29440149

[9] Mourtzakis M Prado CM Lieffers JR Reiman T McCargar LJ Baracos VE. A practical and precise approach to quantification of body composition in cancer patients using computed tomography images acquired during routine care. Appl Physiol Nutr Metab. (2008) 33:997–1006. 10.1139/H08-075 18923576

[10] Shen W Punyanitya M Wang Z Gallagher D St-Onge MP Albu J. Total body skeletal muscle and adipose tissue volumes: estimation from a single abdominal cross-sectional image. J Appl Physiol. (2004) 97:2333–8. 10.1152/japplphysiol.00744.2004 15310748

[11] Popuri K Cobzas D Esfandiari N Baracos V Jagersand M. Body composition assessment in axial CT images using FEM-based automatic segmentation of skeletal muscle. IEEE Trans Med Imaging. (2016) 35:512–20. 10.1109/TMI.2015.2479252 26415164

[12] Miller KD Jones E Yanovski JA Shankar R Feuerstein I Falloon J. Visceral abdominal-fat accumulation associated with use of indinavir. Lancet. (1998) 351:871–5. 10.1016/S0140-6736(97)11518-5 9525365

[13] Mitsiopoulos N Baumgartner RN Heymsfield SB Lyons W Gallagher D Ross R. Cadaver validation of skeletal muscle measurement by magnetic resonance imaging and computerized tomography. J Appl Physiol. (1998) 85:115–22. 10.1152/jappl.1998.85.1.115 9655763

[14] Stene GB Balstad TR Leer ASM Bye A Kaasa S Fallon M. Deterioration in muscle mass and physical function differs according to weight loss history in cancer cachexia. Cancers (Basel). (2019) 11:1925. 10.3390/cancers11121925 31816924 PMC6966581

[15] Agustsson T Ryden M Hoffstedt J van Harmelen V Dicker A Laurencikiene J. Mechanism of increased lipolysis in cancer cachexia. Cancer Res. (2007) 67:5531–7. 10.1158/0008-5472.CAN-06-4585 17545636

[16] Lakens D. Calculating and reporting effect sizes to facilitate cumulative science: a practical primer for t-tests and ANOVAs. Front Psychol. (2013) 4:863. 10.3389/fpsyg.2013.00863 24324449 PMC3840331

[17] Cohen J Statistical Power Analysis for the Behavioral Sciences, 2nd edn. New York, NY: Taylor & Francis Inc (1988).

[18] Prado CM Purcell SA Alish C Pereira SL Deutz NE Heyland DK. Implications of low muscle mass across the continuum of care: a narrative review. Ann Med. (2018) 50:675–93. 10.1080/07853890.2018.1511918 30169116 PMC6370503

[19] Faber J Fonseca LM. How sample size influences research outcomes. Dental Press J Orthod. (2014) 19:27–9. 10.1590/2176-9451.19.4.027-029.ebo 25279518 PMC4296634

[20] MacDonald AJ Greig CA Baracos V. The advantages and limitations of cross-sectional body composition analysis. Curr Opin Support Palliat Care. (2011) 5:342–9. 10.1097/SPC.0b013e32834c49eb 21986910

[21] Baracos V Kazemi-Bajestani SM. Clinical outcomes related to muscle mass in humans with cancer and catabolic illnesses. Int J Biochem Cell Biol. (2013) 45:2302–8. 10.1016/j.biocel.2013.06.016 23819995

[22] Shen W Chen J Gantz M Velasquez G Punyanitya M Heymsfield SB. A single MRI slice does not accurately predict visceral and subcutaneous adipose tissue changes during weight loss. Obesity (Silver Spring, Md). (2012) 20:2458–63. 10.1038/oby.2012.168 22728693 PMC3466347

[23] Dobs AS Boccia RV Croot CC Gabrail NY Dalton JT Hancock ML Effects of enobosarm on muscle wasting and physical function in patients with cancer: a double-blind, randomised controlled phase 2 trial. Lancet Oncol. (2013) 14:335–45. 10.1016/S1470-2045(13)70055-X 23499390 PMC4898053

[24] Temel JS Abernethy AP Currow DC Friend J Duus EM Yan Y. Anamorelin in patients with non-small-cell lung cancer and cachexia (ROMANA 1 and ROMANA 2): results from two randomised, double-blind, phase 3 trials. Lancet Oncol. (2016) 17:519–31. 10.1016/S1470-2045(15)00558-6 26906526

[25] Garcia JM Boccia RV Graham CD Yan Y Duus EM Allen S. Anamorelin for patients with cancer cachexia: an integrated analysis of two phase 2, randomised, placebo-controlled, double-blind trials. Lancet Oncol. (2015) 16:108–16. 10.1016/S1470-2045(14)71154-4 25524795

[26] Ramage MI Skipworth RJE. The relationship between muscle mass and function in cancer cachexia: smoke and mirrors? Curr Opin Support Palliat Care. (2018) 12:439–44. 10.1097/SPC.0000000000000381 30138131

[27] Tsoli M Robertson G. Cancer cachexia: malignant inflammation, tumorkines, and metabolic mayhem. Trends Endocrinol Metab. (2013) 24:174–83. 10.1016/j.tem.2012.10.006 23201432

[28] Daas SI Rizeq BR Nasrallah GK. Adipose tissue dysfunction in cancer cachexia. J Cell Physiol. (2018) 234:13–22. 10.1002/jcp.26811 30078199

[29] Maeda N Funahashi T Matsuzawa Y Shimomura I. Adiponectin, a unique adipocyte-derived factor beyond hormones. Atherosclerosis. (2020) 292:1–9. 10.1016/j.atherosclerosis.2019.10.021 31731079

[30] Kerem M Ferahkose Z Yilmaz UT Pasaoglu H Ofluoglu E Bedirli A. Adipokines and ghrelin in gastric cancer cachexia. World J Gastroenterol. (2008) 14:3633–41. 10.3748/wjg.14.3633 18595130 PMC2719226

[31] Diakowska D Markocka-Maczka K Szelachowski P Grabowski K. Serum levels of resistin, adiponectin, and apelin in gastroesophageal cancer patients. Dis Markers. (2014) 2014:619649. 10.1155/2014/619649 25049439 PMC4094727

[32] Smiechowska J Utech A Taffet G Hayes T Marcelli M Garcia JM. Adipokines in patients with cancer anorexia and cachexia. J Investig Med. (2010) 58:554–9. 10.2310/JIM.0b013e3181cf91ca 20215915

[33] von Frankenberg AD Silva FM de Almeida JC Piccoli V do Nascimento FV Sost MM Effect of dietary lipids on circulating adiponectin: a systematic review with meta-analysis of randomised controlled trials. Br J Nutr. (2014) 112:1235–50. 10.1017/S0007114514002013 25192422

[34] Wu JH Cahill LE Mozaffarian D. Effect of fish oil on circulating adiponectin: a systematic review and meta-analysis of randomized controlled trials. J Clin Endocrinol Metab. (2013) 98:2451–9. 10.1210/jc.2012-3899 23703724 PMC3667269

[35] Abbott JH. The distinction between randomized clinical trials (RCTs) and preliminary feasibility and pilot studies: what they are and are not. J Orthop Sports Phys Ther. (2014) 44:555–8. 10.2519/jospt.2014.0110 25082389

[36] Bland JM Altman DG. Comparisons against baseline within randomised groups are often used and can be highly misleading. Trials. (2011) 12:264. 10.1186/1745-6215-12-264 22192231 PMC3286439

